# The Relative Importance of Janzen-Connell Effects in Influencing the Spatial Patterns at the Gutianshan Subtropical Forest

**DOI:** 10.1371/journal.pone.0074560

**Published:** 2013-09-05

**Authors:** Yan Zhu, Stephan Getzin, Thorsten Wiegand, Haibao Ren, Keping Ma

**Affiliations:** 1 State Key Laboratory of Vegetation and Environmental Change, Institute of Botany, Chinese Academy of Sciences, Beijing, China; 2 Department of Ecological Modelling, UFZ Helmholtz Centre for Environmental Research, Leipzig, Germany; The University of Texas at San Antonio, United States of America

## Abstract

The Janzen-Connell hypothesis is among the most important theories put forward to explain species coexistence in species-rich communities. However, the relative importance of Janzen-Connell effects with respect to other prominent mechanisms of community assembly, such as dispersal limitation, self-thinning due to competition, or habitat association, is largely unresolved. Here we use data from a 24-ha Gutianshan subtropical forest to address it. First we tested for significant associations of adults, juveniles, and saplings with environmental variables. Second we evaluated if aggregation decreased with life stage. In a third analysis we approximately factored out the effect of habitat association and comprehensively analyzed the spatial associations of intraspecific adults and offspring (saplings, juveniles) of 46 common species at continuous neighborhood distances. We found i) that, except for one, all species were associated with at least one environmental variable during at least one of their life stages, but the frequency of significant habitat associations declined with increasing life stage; ii) a decline in aggregation with increasing life stage that was strongest from juveniles to adults; and iii) intraspecific adult-offspring associations were dominated by positive relationships at neighborhood distances up to 10 m. Our results suggest that Janzen-Connell effects were not the dominant mechanisms in structuring the spatial patterns of established trees in the subtropical Gutianshan forest. The spatial patterns may rather reflect the joint effects of size-dependent self-thinning, dispersal limitation and habitat associations. Our findings contribute to a more comprehensive understanding of the relative importance of Janzen-Connell effects in influencing plant community structure under strong topographic heterogeneity.

## Introduction

Numerous mechanisms have been proposed to explain the maintenance of diversity in species-rich plant communities (e.g. [1], [2], [3], [4]). It is well known that the habitat niches alone cannot explain the high species richness of tropical forests [5]. Therefore, other mechanisms have been proposed to explain the high biodiversity of the tropics, like those described by the Janzen-Connell hypothesis (J-C) [6], [7]. This hypothesis predicts that the probability of offspring survival should increase with the distance from conspecific adults, and decrease with conspecific offspring density, due to predation by host-specific pests near the adults. Nathan and Casagrandi [8] showed that a hump-shaped J-C pattern can occur if the mean distance over which predators are active is lower than that over which seeds are dispersed, and a declining pattern in offspring density (with maximum close to the parent trees) occurs when predation distances equal or exceed that of dispersal.

Due to the J-C effects species patterns should become more regular (or less aggregated) with increasing life stage, and in case of a hump-shaped pattern, younger life stages should have their peak closer to adults [6], [7], [9]. The thinning of offspring near adults leaves space and resources for the establishment of other species leading to interspecific mingling (i.e., individuals of different species co-occur frequently together at smaller areas), thereby contributing to the coexistence of multiple species [4]. It is well established that J-C effects influence the fate of seeds and seedlings, and negatively regulate population structure across different latitudes, especially in tropical areas [4], [10], [11], [12], 13. However, the outcome of subtle J-C effects for larger size classes that may accumulate in the long run is difficult to detect directly through short-term experiments and observations [4], [9], [14].

Given that the majority of seeds are dispersed in natural forests near parent trees [15], distance and density-dependent survival should result in a peak of offspring density at an intermediate distance from conspecific adults [6]. The J-C effects have, by definition, a strong spatial component and should therefore leave identifiable spatial patterns of plant locations that could be detected with spatial point pattern analysis [9], [16], [17], [18]. A detailed analysis of the multivariate spatial point pattern of a plant community at multiple levels of organization may therefore reveal a signal of long-term accumulated J-C effects [19]. To test the prediction of the density-dependent component of the J-C hypothesis (i.e., species aggregation decreases with life stage; e.g., [9]) one may study changes in the spatial pattern of species with life stage (sapling, juvenile, adult). To test the distance-dependent component of the J-C hypothesis (i.e., the spatial association of offspring around adult trees should become weaker with increasing life stage) one may study changes in the spatial association of saplings around adults relative to that of juveniles around adults (e.g. [18], [19]).

However, habitat associations may mask the signal of J-C effects [20], [21], [22], [23]. For example, higher mortality of juveniles in less suitable areas may lead to a shrinking of the spatial distribution of the species and increasing aggregation with increasing life stage [24], [25]. Additionally, if small trees that are typically distributed in clusters increase in size, they begin to compete with their immediate neighbors for limited space and nutrients (i.e., self-thinning; [22], [23], [26]). Self-thinning at the transition from juvenile to adult may therefore imprint a spatial signature similar to that of the density-dependent component of the J-C hypothesis [22], [23], [26], [27].

In this study we used the data of trees with diameter at breast height (dbh)≥1 cm in a 24-ha fully mapped subtropical Gutianshan forest dynamic plot (hereafter Gutianshan FDP; [28], [29], [30]) to assess potential signals of accumulated J-C effects from the analysis of spatial patterns. To put J-C effects into perspective, we tested in a first analysis for significant association of different species with environmental variables. In a second analysis we studied changes in intraspecific patterns with life stage. We expect a decrease of aggregation with life stage due to J-C effects and self-thinning [9], [12]. In a third analysis we focus on the intraspecific spatial association of offspring to adult trees by quantifying the density of saplings and juveniles at different neighborhoods around adults. The J-C hypothesis predicts fewer significant and positive juvenile-adult associations than sapling-adult associations, and at an intermediate distance from conspecific adults, saplings should have their maximum density peak closer to adults than juveniles [8], [31], [32].

Departures from the expectations may occur if J-C effects were not present or if they were overpowered by other processes or mechanisms such as competition [33] or smaller-scale gradients in habitat association. Analyzing the spatial patterns of the tree community at the Gutianshan FDP plot at multiple levels of organization allows us to determine if J-C effects play a major role in shaping spatial community patterns in this forest relative to other processes.

## Methods

### Study Site and Data Collection

No specific permits were required for the described field studies in and outside of Gutianshan National Nature Reserve. The nature reserve is owned and managed by the state and its government and the location including the site for our sampling are not privately-owned or protected in any way. So, specific permission for non-profit research is not required. The field studies did not involve in endangered or protected species in this area.

The Gutianshan FDP (29°10′19′′–29°17′41′′ N and 118°03′49′′–118°11′12′ E) is located in the Gutianshan National Nature Reserve in eastern China. The region is subtropical, with a mean annual precipitation of 1963.7 mm and mean annual temperature of 15.3°C. The shape of the study plot is a rectangle of 600 m length and 400 m width. Within this plot, 140,700 woody stems ≥1 cm dbh were counted when the subtropical evergreen broadleaf forest was first censused in 2005. These woody species included 49 families, 103 genera and 159 species. Fagaceae, Lauraceae, Theaceae, and Magnoliaceae are the dominant families [34]. The Gutianshan FDP is very rugged with elevation varying from 446.3 to 714.9 m above sea level and can be divided into five topographic habitat types that are basically defined by valleys and ridges [25], [28]. Interactions of fire disturbance with topography caused some of the differences in species composition among habitat types and tree richness and composition was controlled by spatially structured habitat [28].

Species were classified into three growth forms: shrubs (<5 m), under-story trees (≥5 and <15 m), and canopy trees (≥15 m), according to their typical maximum height. To meet the needs of statistical analysis, we tested the spatial distributions of 46 species with ≥40 individuals at each life history stage. These species made up 89.7% of the total stems in the plot. To detect possible differences between species belonging to different height strata we defined size classes of saplings, juveniles and adults separately for shrubs, under-story, and canopy trees (Table 1).

**Table 1:** The classification of saplings, juveniles and adults based on the size classes of dbh (cm; diameter at breast height) separately for shrubs, under-story, and canopy trees.

Growth forms	Saplings	Juveniles	Adults
Canopy trees	1–5	5–10	≥10
Under-story trees	1–2.5	2.5–5	≥5
Shrubs	1–1.5	1.5–2	≥2

### Point Pattern Analysis

#### Analysis 1: The relationship of species distribution patterns to topographic factors

We test if the spatial distribution patterns of adults, juveniles, and saplings of a given species depended on topographic habitat variables. Quantification of habitat association allows us to assess the degree to which J-C effects might be masked by habitat association and to interpret our results. To this end we employed the Berman test [35]. The test compares the mean *S*_obs_ of the covariate values *v*(**x**_i_) at the locations **x**_i_ of the trees of a given size class d to corresponding values *S*_sim_ generated by repeated simulations from a suitable null model. Significant departures from the null model are assessed by the test statistic *Z*_1_ =  (*S*_obs_−*μ*)/*σ* where *μ* is the mean value of *S*_sim_ under the null model and *σ* ^2^ the corresponding variance [35]. The null distribution of this test statistic is approximately the standard normal distribution.

The null model should randomize the observed pattern in a way that it is independent of the covariate but maintains its observed clustering [34]. Tests based on a null model that does not account for the observed clustering will be more likely to reject the null hypothesis erroneously. We generated null distributions of the observed species pattern based on the non-parametric technique of pattern reconstruction [36], [37] that is able to generate stochastic replicates of the observed pattern that approximate several summary statistics of the observed pattern very well (i.e., pair correlation function, *K*-function, spherical contact distribution, nearest neighbor distribution functions). The used pattern reconstruction algorithm described in Wiegand et al. [36] is a variation of simulated annealing that generates by trial and error a series of patterns that approach in each simulation step the summary statistics of the observed patterns more closely. The statistical properties of the observed pattern are measured by several functional summary statistics. The pattern reconstruction algorithm therefore generates stochastic replicates of the observed pattern required for the null model.

We conducted the Berman test for the covariates mean elevation, mean slope, and mean convexity in 20×20 m quadrats as environmental variables. Detailed definitions about how to obtain the mean elevation, mean slope, and mean convexity can be found in Legendre et al. [28] and Figure S1.

#### Analysis 2: change of intraspecific patterns with life stage

In this analysis we tested if the spatial pattern of a given species becomes more regular with progressing life stage. For this purpose we used the recently developed K2-function which has the interesting property that it can reveal regularity and aggregation despite presence of (larger-scale) gradients in environmental conditions (Fig. S2; [38]).

The K2-function is derived from the pair-correlation function *g*(*r*). The *g*(*r*) is the mean density of points at distance *r* away from the points of the pattern, divided by the overall density λ of points within the study area (i.e., λ = number of points/area) [16], [37]. Thus, the pair-correlation function is a relative neighborhood density function with a value of one if the neighborhood density equals the overall intensity λ (i.e., a random pattern), and values larger (or smaller) than one if the neighborhood density around points of the pattern is larger (or smaller) than the intensity λ, respectively. Aggregation at distance *r* is indicated by *g*(*r*)>1 and regularity by *g*(*r*)<1. Once the pair-correlation function is estimated, the K2-function describes the expected change in relative neighborhood densities over a small range of distances (i.e., from r−Δr to r+Δr):
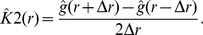
(1)


The pair-correlation function that basically measures the neighborhood density has problems to identify aggregation if the pattern is heterogeneous because both aggregation and heterogeneity cause locally elevated point densities. The underlying rationale of the K2-function is that larger-scale heterogeneity in the intensity function introduces a smooth effect on the neighborhood density in general whereas point interactions that cause aggregation or regularity typically yield more rapid changes in the neighborhood density. That means that the shape of the K2-function (that measures the rate of change in the neighborhood density) should be largely independent on larger-scale heterogeneity and therefore allows for an assessment of regularity or clustering even in the presence of heterogeneity. The K2 therefore indicates the distances *r* where large changes occur from higher to lower aggregation or from higher regularity to lower regularity. The distance intervals of aggregation or regularity can therefore be identified when looking on both, the pair-correlation function and the K2-function. Comparing the observed K2 function to that generated under complete spatial randomness (CSR) allows identification of aggregation or regularity in spite of environmental heterogeneity (see [38] for exhaustive tests of this property of the K2-function). We test for significant departures from the CSR null model using the 5th-lowest and 5th-highest value of 199 Monte Carlo simulation envelopes estimated for the 2–50 m distance interval. Values of *K*2(*r*) above or below the simulation envelopes indicate regularity or aggregation of the pattern, respectively [38].

Because simulation envelopes test at several spatial distances *r* simultaneously, type I error may occur (i.e., the null model may be rejected even if it is true; [39]). We therefore combined the common simulation envelope method with a goodness-of-fit test (GoF; [30], [39], [40], [41]) that provides a *p*-value for significant differences between the data and the null model over all spatial distances considered.

#### Analysis 3: Intraspecific spatial associations between different life stages

We used bivariate pair-correlation function *g*_12_(*r*) [16], [37], [42], [43], [44] to test for positive and negative spatial associations between different size classes of a given species. To reveal a signal of adult-offspring (or juvenile-sapling) association we need a null model that (approximately) conditions on the potentially confounding effects of habitat association which are expected to be present at the Gutianshan plot [30]. Thus, the null model needs to account for potential spatial variation in the intensity function λ_2_(**x**) of the second component pattern (e.g., offspring) that can arise for example if some areas of the plot are less suitable for the species than others, while it keeps the locations of the first component pattern (i.e., adults as an antecedent pattern; Fig. S3; [16]).

Technically, we implemented this null model as a heterogeneous Poisson null model (HP) of the offspring pattern together with a non-parametric estimate of its intensity function [16], [27], [45]. In the heterogeneous Poisson point process the occurrence of a point of the second component pattern is independent of that of others, but the points are distributed in accordance with the intensity function λ_2_(**x**) that varies with location **x**. We used a neighborhood radius of *R* = 30 m as bandwidth of the Epanechnikov kernel [42], [45] because we expect that J-C (and other) interactions may occur predominately within 30 m from the parent trees.

If there is a positive association (attraction) or negative association (repulsion or segregation) between the two patterns, the neighborhood density of pattern 2 at distance *r* from the points of pattern 1 will be higher or lower on average than expected under the null model, respectively. Note that we may also determine the intensity function parametrically using environmental variables (e.g. [9], [46]). However, this approach would work well only in our case if the environmental variables that are available do indeed explain the spatial distribution pattern of the species well. This may not always be the case and renders this approach unsuitable for our particular purpose.

We analyzed the following bivariate patterns: juveniles around adults, saplings around adults, and saplings around juveniles. We used a spatial resolution of 1 m and a ring width of 3 m at distances of 0–50 m. Significant departure from null models was evaluated using the 5th-lowest and 5th-highest value of 199 Monte Carlo simulation envelopes (Fig. S4) and the GoF test. We expected a *p*-value of the GoF test smaller than 0.05 at distances of 0–50 m, which suggests significant small-scale association.

We summarized the main hypotheses and null models applied in point pattern analyses in Table 2. All point pattern analyses were done using the software Programita [16] and software R 2.9.1 [47].

**Table 2:** Main hypotheses, applied point pattern analyses, related illustrations (i.e. figures, tables, and supporting information), and main results rejecting or supporting the main hypotheses in this study.

Hypotheses	Point pattern analyses and null models	Related illustrations	Results
***Analysis 1***			
The distribution of tree species depends onenvironmental variables	The Berman test under the null model of patternreconstruction applied to all adult trees, juveniles,and saplings of a given species	Fig. S1; table S1	Results supported theprediction
***Analysis 2***			
Decrease of aggregation with life stage	The univariate K2-function under the CSR nullmodel applied to saplings, juveniles, and adults	Fig. 1 & S2	Overall, results supportedthe prediction
***Analysis 3***			
(i) Fewer significant and positive juvenile-adultassociations than sapling-adult associations,(ii) a peak of sapling and juvenile neighborhooddensity at an intermediate distance fromconspecific adults, (iii) saplings should have their maximumcloser to adults than juveniles	The bivariate pair-correlation function and aheterogeneous Poisson null model (with maximaldisplacement distance R = 30 m) were used toanalyze the spatial associations of adults to offspring(i.e. juveniles, saplings)	Fig. 2 & S3, S4; table 3	Results rejected thepredictions (i) and (iii);little support for theprediction (ii)

## Results

### Analysis 1: The Relationship of Species Distribution Patterns to Topographic Factors

Except for one, all species were significantly associated to at least one topographic environmental variable with at least one of their life stages (Table S1, examples in Fig. S1). There was a consistent decline in the proportion of species that showed dependency with covariates from saplings to adults. We found that 78% of saplings, 67% of juveniles, and 54% of adults were associated to elevation, respectively, and 83%, 72%, and 65% of saplings, juveniles, and adults, were associated with convexity, respectively. However, slope had a comparatively small effect (30%, 33%, 30%). The decline in the proportion of significant effects with life stage was not primarily caused by abundance effects; the 46 species comprised 60,739 saplings, 32,476 juveniles and 32,979 adults. The rank correlation coefficients between the *Z*_1_ value of the Berman test and the species abundance indicated weak correlations (0.3 for convexity, 0.23 for elevation and 0.2 for slope) and on overall 47% (27 of 57 cases) of species with abundances below 100 individuals and 60% (213 of 357) of species with abundances above 100 individuals showed significant effects. Thus, the results were not mainly driven by species abundance.

Overall we found that 65.2% of the species were associated simultaneously with at least one environmental variable (i.e., either positively or negatively correlated) in the same direction at all three life stages. This illustrates strong dependencies of the species on abiotic habitat niches. Only three species (6.5%) showed an “opposite” behavior when adults were negatively but juveniles and recruits positively associated with a covariate. These species were *Loropetalum chinense, Rhododendron ovatum, and Ternstroemia gymnanthera.*

### Analysis 2: Change of Intraspecific Patterns with Life Stage

**Figure 1:**
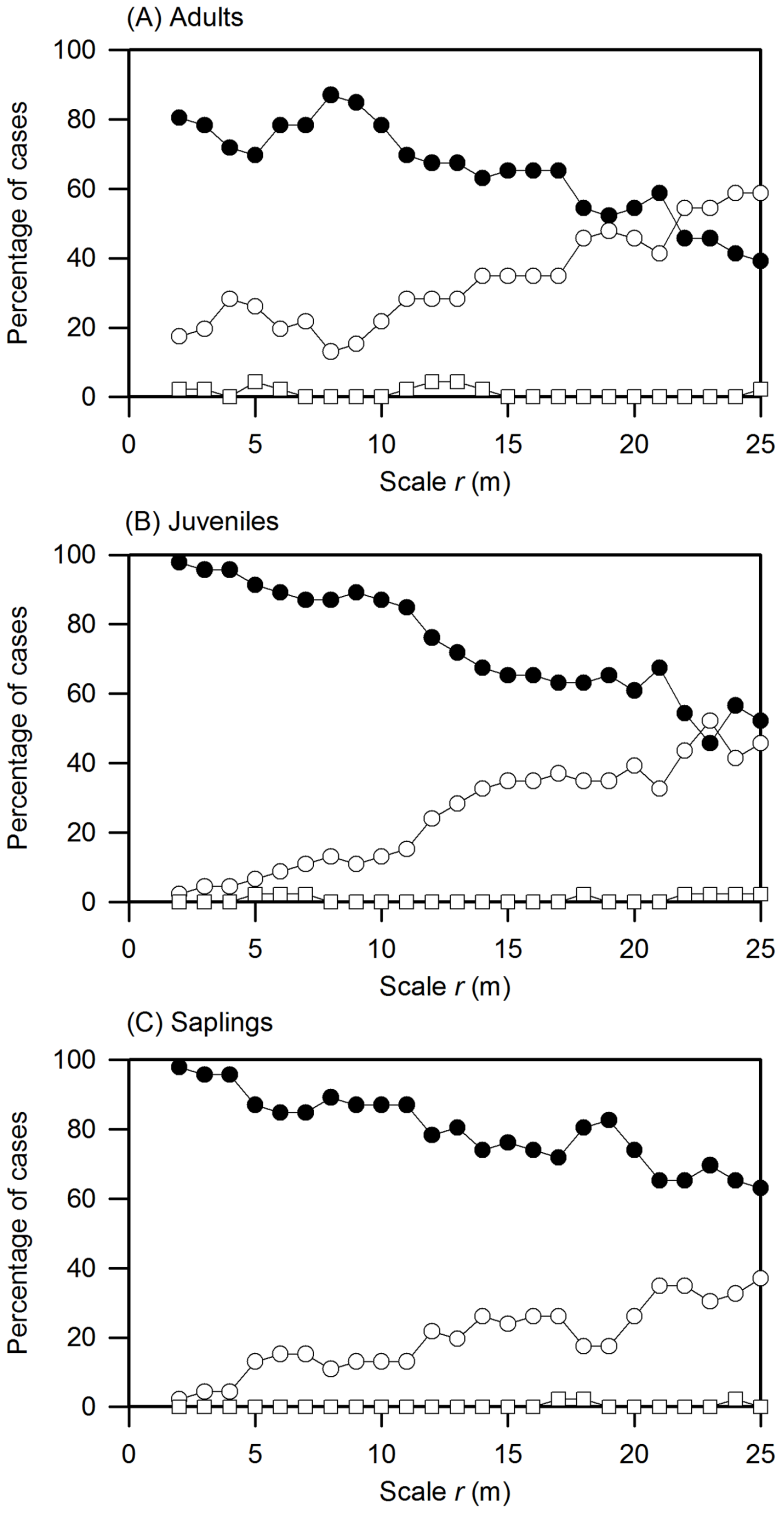
Analysis with the univariate K2-function. Percentage cases in which conspecific adult (A), juvenile (B), and sapling (C) patterns showed significant aggregation (black circles), regularity (squares), and randomness (white circles) at distance *r*. Significant departures from the CSR null model were estimated using the 5th-lowest and 5th-highest value of 199 Monte Carlo simulation envelopes for the 2–50 m distance interval. Values of *K2(r)* above, below or within the simulation envelopes indicate regularity, aggregation or randomness of the pattern, respectively. Note that the K2-function removes the confounding effects of large-scale habitat association.

We evaluated the change of intraspecific patterns with life stage in two different ways with the K2-function (see Fig. S2 for an example). First, we assessed for each scale *r* the percentage of species that showed significant deviation from the null model based on simulation envelopes. For all three life stages, aggregation dominated the patterns up to approximately 25 m, with random distributions dominating thereafter at larger scales (Fig. 1). Regular patterns were rare. Overall, significant cases with small-scale aggregation were more frequent in the juvenile (Fig. 1B) and sapling (Fig. 1C) stages compared with the adult stages (Fig. 1A). These results were confirmed by the goodness-of-fit test. Because we were mainly interested in smaller-scale effects in this analysis, we applied the GoF test for the 1–25 m distance interval. Significant departure from the null model occurred only in 52.2% of the cases for adults, but in 84.8% and 84.4% for juveniles and saplings, respectively.

Second, we determined for each species and distances *r* = 2, 4, 6, 8, and 10 m if intraspecific aggregation declined from sapling (subscript sa) to juvenile (subscript ju) [i.e., *K*2_sa_(*r*)−*K*2_ju_(*r*)<0] and from juvenile to adult (subscript ad) [i.e., *K*2_ju_(*r*)−*K*2_ad_(*r*)], and counted the cases where this occurred. Note that unlike *g*(*r*), the K2-function indicates significant aggregation with negative values [38]. The juvenile-adult transition (i.e., *ju-ad*) yielded at distances of 2–4 m for 76% of all species (35) a decline in intraspecific aggregation, and averaged over all scales in 66% of all cases. The sapling-juvenile transition (i.e., *sa-ju*) yielded at the 2 m distance for 43% of all species (20) a decline in intraspecific aggregation, and averaged over all scales in 46% of all cases. Viewed as average over all three groups of life stages and individual scales, self-thinning occurred most frequently at the small 2 m (62%) and 4 m scales (66%), but was less frequent at larger scales; i.e. 6 m (55%), 8 m (54%), 10 m (50%).

### Analysis 3: Intraspecific Spatial Association between Different Life Stages

The overall spatial associations between different life-history stages showed a strong prevalence of positive effects (Table 3; see Fig. S4 for an example). The GoF test and the simulation envelopes showed that the adult-juvenile association was significant and positive for 36 species (i.e., significant attraction), and the adult-sapling association was significant and positive for 32 species and negative for 1 species. All 46 species showed a significant positive association between juveniles and saplings.

**Table 3:** The number of species with positive, negative or no significant association between different life stages (the percentage follows in parentheses).

Bivariatepattern	Testedspecies	Positive association at 0–50 m^†^ (peak at distance)				No association at0–50 m^†^	Negativeassociationat 0–10 m^†^
		<1 m	≥1 m	≥3 m	≥5 m		
Adults vs. juveniles	36	20 (55.6)	16 (44.4)	8 (22.2)	1 (2.8)	0	0
Adults vs.saplings	33	12 (36.4)	19 (57.6)	14 (42.4)	8 (24.2)	0	4 (12.1)
Juveniles vs.saplings	46	35 (76.1)	11 (23.9)	3 (6.5)	2 (4.3)	0	0

GoF test over distance interval 0–50 m has *p*<0.05.

**Figure 2:**
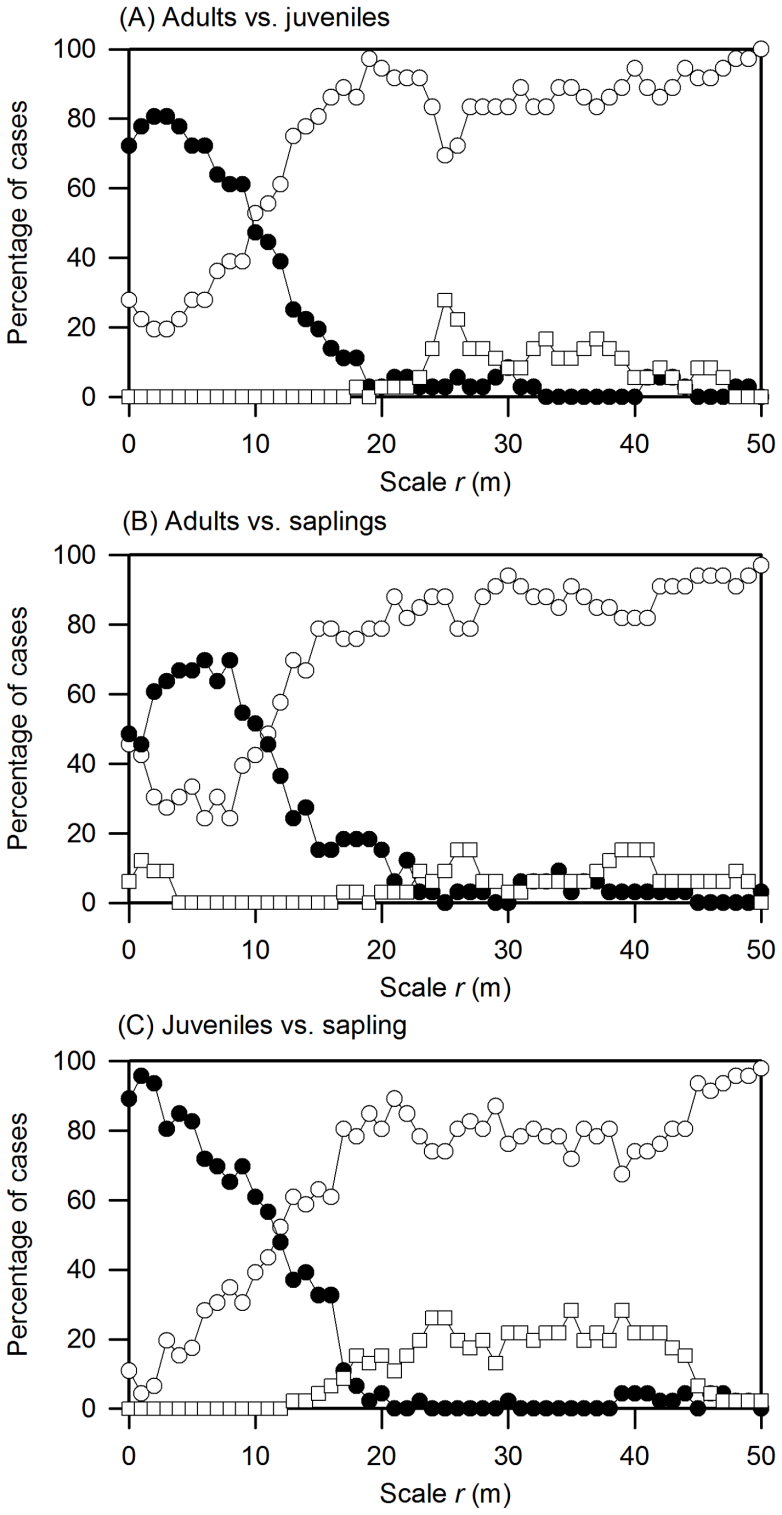
Results of analysis for conspecific spatial association between different life-history stages. Percentage cases in which different life-history stages showed significant attraction (black circles) and repulsion (squares), and no significant association (white circles) at distance *r*. Pattern 1 was the pattern of older trees and pattern 2 that of younger trees.

To obtain a rough estimate of the magnitude of distance-dependent effects, we counted for each distance *r* the number of species (using only species where the GoF test was significant with *p*<0.05) for which the pair-correlation function was above the 5th-highest value of the 199 Monte Carlo simulations (Fig. 2). The frequency of positive associations declined with distance, *r*, and fell below the 5% error rate after distances >20 m. This indicates that the assumption of separation of scales was met in this analysis for positive associations [27], [45]. Negative associations were absent at small distances, but appeared in some cases at distances larger than 20 m (Fig. 2).

The percentage of cases with positive adult-juvenile associations increased up to distances of 2 m and 3 m (80.6%) and then decreased almost linearly beyond that (Fig. 2A). The percentage cases with positive adult-sapling associations peaked at distances of 6 m and 8 m (69.7%) and then decreased (Fig. 2B). Finally, almost all species showed a positive juvenile-sapling association at close distances and the percentage of significant associations decreased almost directly with increasing spatial distances (Fig. 2C). Significant negative associations occurred between adults and saplings at distances smaller than 10 m for only four abundant species (*Castanopsis eyrei*, *Schima superba*, *Eurya muricata*, and *Rododendron ovatum*), but disappeared for adults and juveniles (Fig. 2A, B). Thus, saplings and juveniles were closely mingled together and adults and juveniles showed a tighter spatial association than adults and saplings.

We also determined for each pair of size classes with positive associations the distance *r* with maximal neighborhood density (Table 3). The neighborhood density of juveniles around adults peaked at distances smaller than that of saplings around adults (Table 3). In 12 species we found a peak for sapling density around adults at distances below 1 m, but in 20 species for juveniles (Table 3).The tight juvenile-sapling relationship was confirmed by 35 of 46 species showing the peak in the neighborhood density at distances below 1 m.

## Discussion

We conducted detailed analyses of the complex spatial structures found in a fully mapped 24-ha subtropical forest at Gutianshan FDP, China. To explore the outcome of J-C effects on tree distributions, we contrasted our results to the expectations of spatial structure that would emerge under the J-C hypothesis. Our analyses yielded a clear picture of the spatial structure of the forest at the Gutianshan FDP and outlined a number of patterns. First, the spatial pattern of species are strongly (and mostly consistently among life stages) influenced by topography, but the associations became less frequent with increasing life stage. Second, the degree of aggregation decreased from saplings to adults, and the strongest decline in aggregation occurred at the juvenile-adult transition. Finally, the spatial pattern of offspring showed for most species a strong attraction to the spatial pattern of adult trees, and juveniles showed a tighter association than saplings. Taken these results together, we find that J-C effects are probably not the dominant driving mechanisms in structuring the spatial patterns at the Gutianshan forest. Effects of topographic habitat association may counteract J-C effects, and the decline in aggregation from saplings to adults is more likely to be a result of self-thinning due to competition (for space) because the strongest decline in aggregation occurred at the juvenile-adult transition. The finding that juveniles are more closely associated with adults than saplings to adults is difficult to interpret but may be a result of associations to smaller scale habitat features or saplings being located in suboptimal habitat farther away from adults.

We found surprisingly strong and positive associations between adults and offspring. These results suggest that offspring readily occur near conspecific adults. Indeed, a recent study found that seed density in the Gutianshan plot was closely related with conspecific adult density, and the majority of species showed strong seed dispersal limitation [48]. Additionally, 2/3 of all species were at all three life stages simultaneously and in the same direction associated with (at least) one habitat variable, with saplings showing the highest incidence of habitat association (78% with elevation and 83% with convexity) and adults the lowest (still 54% with elevation and 65% with convexity). Strong species dependency on habitat has also been confirmed in previous studies at Gutianshan [25], [49] and the Sinharaja plot in Sri Lanka that also shows strong topographic structuring [50], but seems stronger than that observed at the BCI tropical forests with little topographic structuring [51], [52], [53]. Thus, seeds falling close to parents were also most likely located in favorable habitat [5], [54]. Here, though habitat effects and dispersal limitation cannot be clearly differentiated in influencing the local patterns, the two joint effects have a strong potential to override J-C effects.

The fact that habitat dependency and the overall strength of environmental filtering is decreasing with the age group from saplings to adults may have the following reasons. Our hypothesis is that the stronger density-dependent mortality at the transition from juveniles to adults leads to a thinning independent on habitat and therefore to a weakening of habitat associations. This result is consistent with previous work in the Gutianshan forest plot [25]. Saplings and juveniles may have stronger direct dependency on habitat because they are less resistant against adverse habitat conditions. In agreement with Kanagaraj et al. [55], our hypothesis is that habitat-specific mortality resulted in high densities of juveniles in their preferred abiotic habitat, but that negative density dependence at the transition to reproductive individuals outweighed the benefits of the optimal seedling habitat and led to adult trees no longer being so strongly associated with that habitat.

Unexpectedly, the positive associations between juveniles and adults were more frequent than those between adults and saplings (Fig. 2) and stronger (i.e., juvenile neighborhood density peaked at shorter distances around adults than sapling neighborhood density). The strongest decline in aggregation occurred at the juvenile-adult transition. This indicates that saplings located close to adults have initially an advantage in the transition to juveniles but when they grow larger they will suffer competition from adults (and close by juveniles) which makes their patterns less aggregated. An example for this finding is given in Fig. S4. It also illustrates how the heterogeneous Poisson null model factored out the strong (habitat driven) larger-scale association of the different life stages of *Distylium myricoides* at distances larger than 30 m (here the pair-correlation function is inside the simulation envelopes) and how it reveals the additional positive effects at distances below 20 m. However, we cannot exclude the possibility that associations to habitat features at smaller scales (i.e.,<20 m) contributed to this. This could explain the unexpectedly tight juvenile-adult association; adults are located in the best habitat and saplings in suboptimal habitat die at the transition to juveniles. Regardless of the explanation for the observed intraspecific association patterns among life stages, they are opposed to expectations from the distance-dependent part of the J-C hypothesis. Because juveniles and saplings appeared to be merged in the same patches close to adults, we find little evidence that J-C effects would make the immediate (<20 m) neighborhood of adults unsuitable for saplings and juveniles.

The existence of strong density-dependent thinning is not surprising given the strong aggregation of the species found at the Gutianshan plot [30] and has been found also in previous studies. For example, Zhu et al. [30] showed that the aggregation of most species at the plot declined from the sapling to the juvenile stage. Similar results were found in the study by Chen et al. [29] based on seedling dynamics of survival and mortality in the same plot. Especially the finding that the strongest decline in aggregation occurred at the juvenile-adult transition suggests that this density-dependent self-thinning may be the result of competition for space but not induced by classical J-C effects. In support of this hypothesis we found in our second analysis that the decline in aggregation at the transition from juveniles to adults was clearly stronger than that from saplings to juveniles. Because the transition from juveniles to adults involves a substantial increase in size we hypothesize that the decrease in aggregation is a result of density-dependent mortality due to competition for space. On the other hand, J-C effects are mostly found for smaller trees and often for seedlings [4] though the J-C effects could be playing a role for larger size individuals in stand development, e.g. as in the case of *Ocotea whitii* at BCI plot [56]. Older plants may be less susceptible to pathogens and have more resources to fend off the effects of herbivores and specialized pathogens [12]. Since conspecific saplings and adults have looser association than conspecific juveniles and adults, it is possible to get some evidence of J-C effects if we test the pattern change at the transition from seedlings to saplings. In addition, as shown in Figures 1A, S2B, conspecific neighbors were regulated by density-dependent thinning mainly at conspecific neighbor distances of <5 m and less at 5–15 m distances. Our finding can serve as a guideline for planning conspecific neighbor distances for subtropical forest recovery and cultivation.

While density-dependent thinning reduced the degree of aggregation, as shown by using the K2-function, only adults of the four species *Castanopsis eyrei*, *Quercus serrata*, *Symplocos stellaris*, and *Vaccinium bracteatum* showed small-scale regularity with K2 analysis. The species *C. eyrei* is with 12,332 individuals the most abundant species at the plot, and exhibited strong density-dependent thinning at distances of 1–30 m from saplings to juveniles [30]. The pioneer species *Q. serrata* was strongly positively associated with convexity which could be explained by its demand for light. Strong competition of this light demanding species with conspecific neighbors may yield the regularity at the adult stage. In contrast, adults of the pioneer species *V. bracteatum* were not significantly associated with convexity or elevation (only with slope) but Zhang et al. [57] showed that this species was associated with soil nutrients.

## Conclusions

In this study we analyzed the multivariate spatial point patterns observed at the Gutianshan forest at multiple levels of organization to assess the relative importance of (long-term accumulated) J-C effects with respect to dispersal limitation, self-thinning due to competition, and habitat association. The results of our spatial analysis for saplings, juveniles and adults did not support expectations derived from the J-C hypothesis. Rather it appears that habitat association together with dispersal limitation produced for most species a tight sapling-adult and juvenile-adult association, and that the strongest self-thinning occurred at the transition to adults, which however did not fully remove aggregation in adults. Dispersal limitation in concert with largely consistent habitat associations across life stages fostered the pattern that offspring readily occurred near conspecific adults. This has the advantage that seeds falling close to parents were also most likely located in favorable habitat, but this tight association was not removed by J-C effects. Our findings contribute to a more comprehensive understanding of the relative importance of J-C effects in influencing the spatial community structure of a subtropical forest subject to strong topographic heterogeneity.

## Supporting Information

Figure S1 Example for Berman test.
(PDF)

Figure S2 Use of the K2-function to quantify decline in aggregation with increasing life stage despite heterogeneity.
(PDF)

Figure S3 The heterogeneous Poisson process and dispersal limitation.
(PDF)

Figure S4 Examples for analyses of the association between different life stages for the species *Distylium myricoides*.
(PDF)

Table S1 Growth forms and abundance of 46 species and the results of analysis 1.
(PDF)
