# Supplementary material for: The Relative Importance of Janzen-Connell Effects in Influencing the Spatial Patterns at the Gutianshan Subtropical Forest
Source: PLoS One. 2013 Sep 5;8(9):e74560. doi: 10.1371/journal.pone.0074560 (PMC3764046; doi:10.1371/journal.pone.0074560)
Supplement: Figure S1 — Example for Berman test. (PDF) [file pone.0074560.s001.pdf]

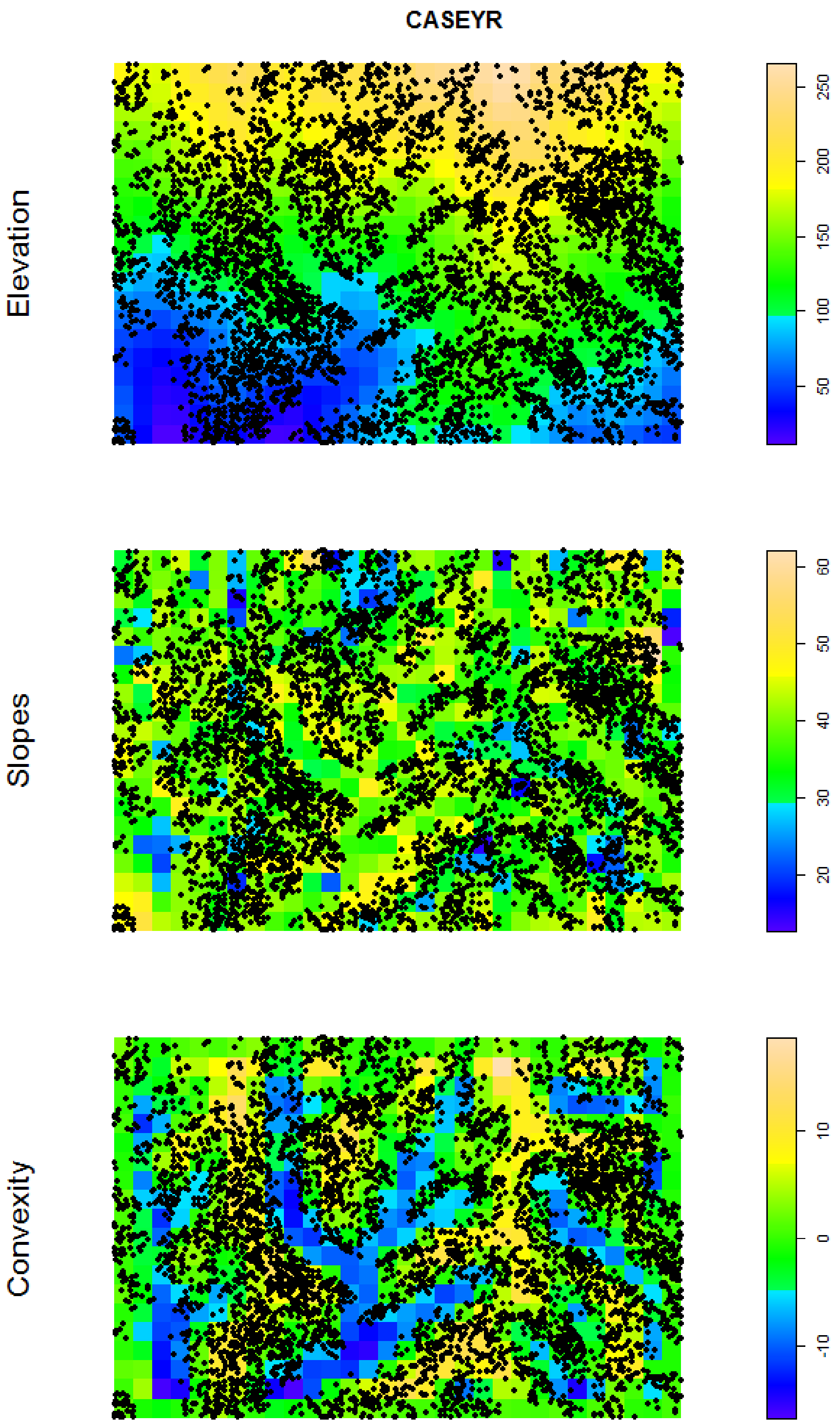

Figure S1. Example for Berman test. Shown are the three covariates elevation (m), slope (degree), and convexity at a grid of 20 m × 20 m and the distribution of adult trees of the species *Castanopsis eyrei* (CASEYR).

Positive values of convexity indicate convex, conversely, negative values indicate concave topography. These three covariates were quantified for each 20 m × 20 m subplot. Mean elevation was the average of the elevation values of four corners of a quadrat. Slope was obtained by direct measure in the plot, which was the average angle deviation from horizontal of each of four triangular planes. Mean convexity was calculated from the measured elevation data. The elevation of a quadrat minus the mean elevation of the eight surrounding quadrats was mean convexity. If the quadrats were at the edge of the plot, convexity of a quadrat was the elevation of the center point minus the mean of the four corners. According to the Berman test, adults of the species *Castanopsis eyrei*were significantly associated with convexity (Z_1 = 7.03; $P < 0.01$) but not with elevation (Z_1 = 0.1) and slope (Z_1 = 0.697). The positive Z_1-value of 7.03 indicates that *C. eyrei* adults prefer those habitats that are characterized by strong convexity.
