# Supplementary material for: The Relative Importance of Janzen-Connell Effects in Influencing the Spatial Patterns at the Gutianshan Subtropical Forest
Source: PLoS One. 2013 Sep 5;8(9):e74560. doi: 10.1371/journal.pone.0074560 (PMC3764046; doi:10.1371/journal.pone.0074560)
Supplement: Figure S2 — Use of the K2-function to quantify decline in aggregation with increasing life stage despite heterogeneity. (PDF) [file pone.0074560.s002.pdf]

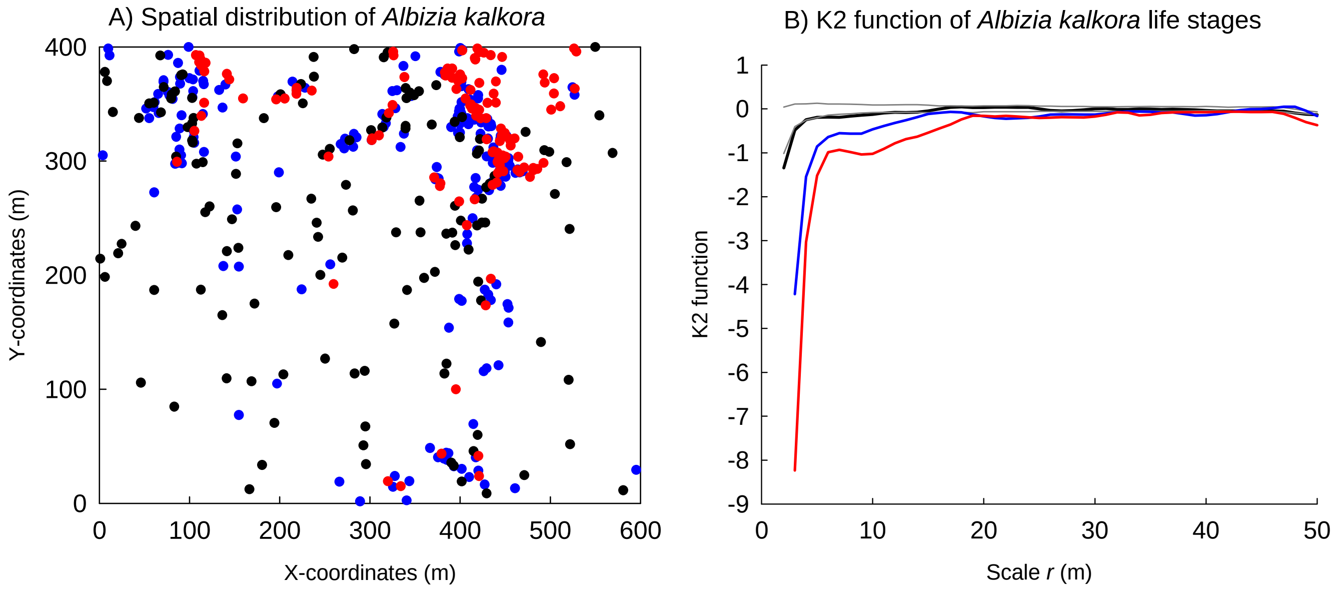

Figure S2. Use of the K2 fucntion to quantify decline in aggregation with increasing life stage despite of heterogeneity. (A) Example of the spatial patterns of adults (black), juveniles (blue), and saplings (red) of the species *Albizia kalkora*. (B) The corresponding K2 functions for adults (black), juveniles (blue), and saplings (red). The grey lines give the simulation envelopes of the 5th-lowest and 5th-highest values of the 199 Monte Carlo simulations under the CSR null model for the adult pattern. Larger negative values of the K2 function indicate stronger aggregation. In the example, the aggregation of the species *A. kalkora* declines from saplings, to juveniles and adults, especially at small neighborhood distances of below 7m.
