# Supplementary material for: The Relative Importance of Janzen-Connell Effects in Influencing the Spatial Patterns at the Gutianshan Subtropical Forest
Source: PLoS One. 2013 Sep 5;8(9):e74560. doi: 10.1371/journal.pone.0074560 (PMC3764046; doi:10.1371/journal.pone.0074560)
Supplement: Figure S3 — The heterogeneous Poisson process and dispersal limitation. (PDF) [file pone.0074560.s003.pdf]

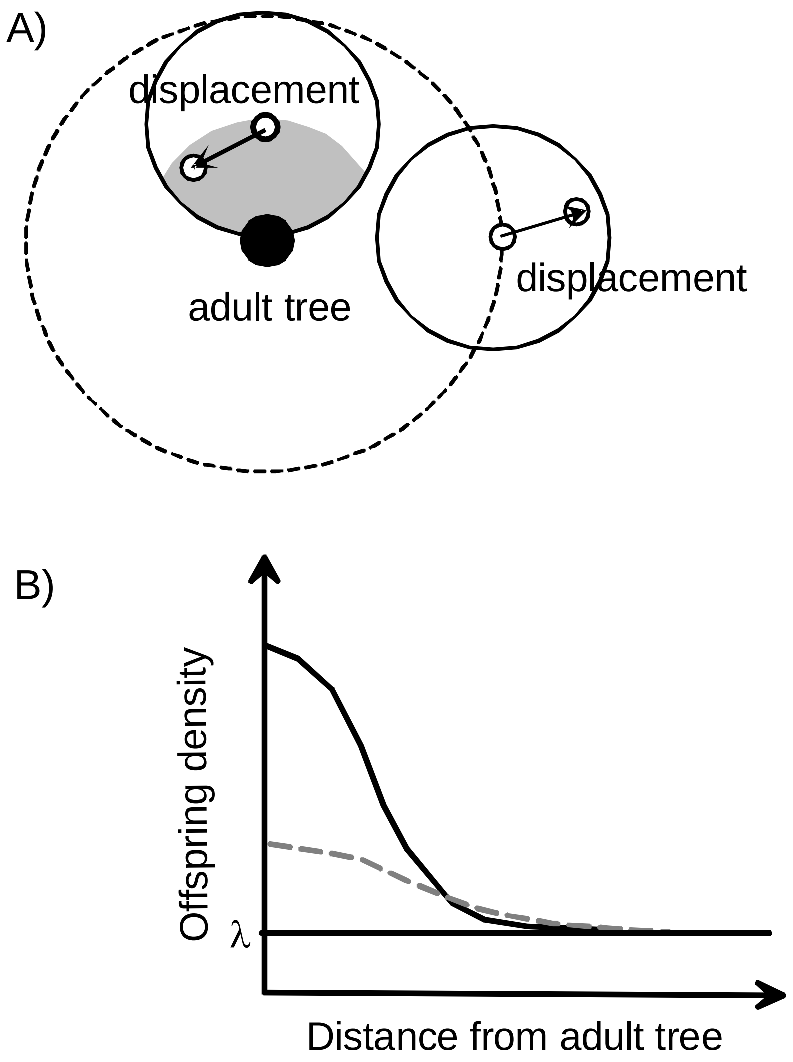

Figure S3. The heterogeneous Poisson process and dispersal limitation. (A) Shown is one adult tree (closed circle) and the approximate range of its dispersal kernel (dashed circle). The open circles show the displacement neighborhood *R* of the heterogeneous Poisson null model and two displacements of offspring points (small open circles, arrows). (B) The mean offspring density at distance *r* from adult trees corresponding to the dispersal kernel (solid line) and the mean offspring density resulting from the displacement of the

heterogeneous Poisson null model. Random relocation of the offspring within the displacement neighborhood will result in most cases in a larger adult-offspring distance compared with the observed adult-offspring distance (only distances within the grey shaded areas will be shorter). The smaller the observed adult-offspring distance, the larger this effect. This will substantially reduce offspring density at short distances r (and slightly increases its value at larger distances) and remove most of the positive association. As a consequence, the signal of dispersal limitation will be detected by the heterogeneous Poisson null model if the displacement neighborhood R is not very small.
