# Supplementary material for: The Relative Importance of Janzen-Connell Effects in Influencing the Spatial Patterns at the Gutianshan Subtropical Forest
Source: PLoS One. 2013 Sep 5;8(9):e74560. doi: 10.1371/journal.pone.0074560 (PMC3764046; doi:10.1371/journal.pone.0074560)
Supplement: Figure S4 — Examples for analyses of the association between different life stages for the species Distylium myricoides. (PDF) [file pone.0074560.s004.pdf]

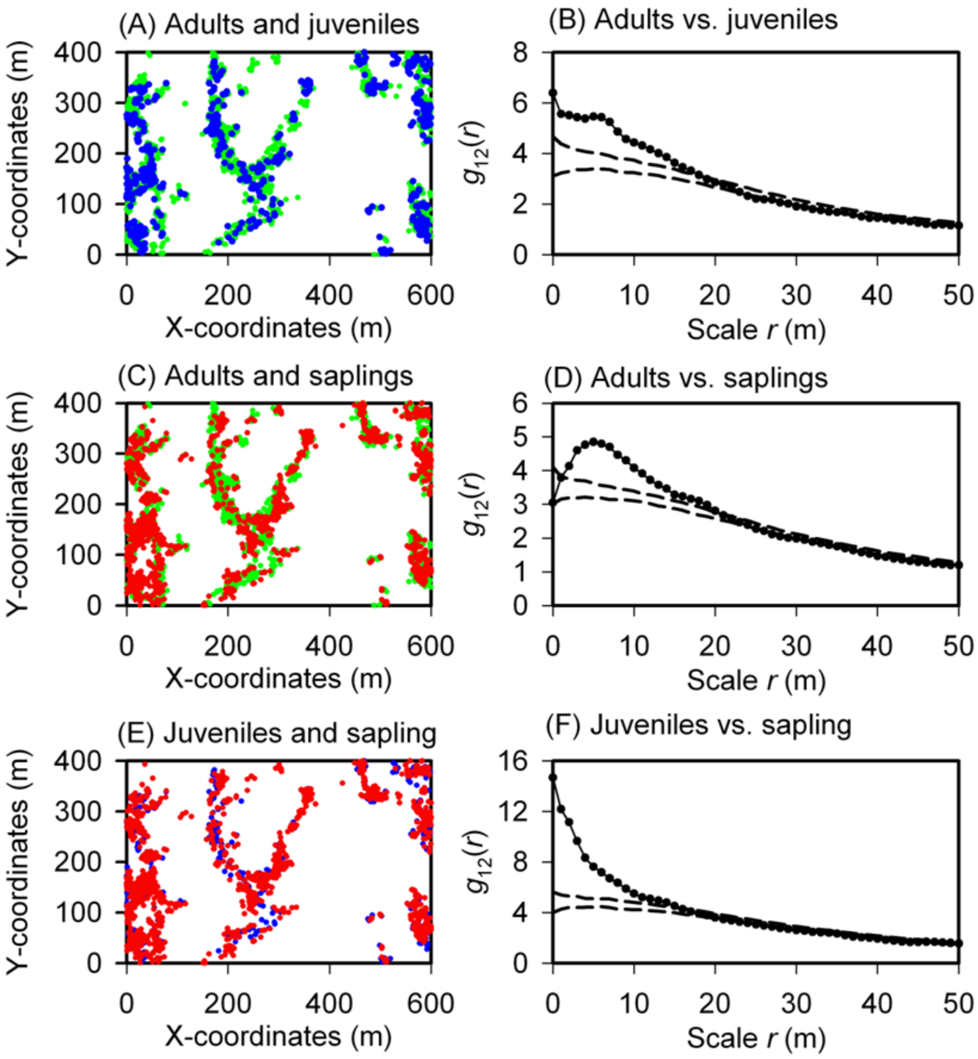

Figure S4. Examples for analyses of the association between different life stages for the species *Distylium myricoides*. The bivariate pair-correlation function $g_{12}(r)$ was contrasted to the heterogeneous Poisson null model. The simulation envelopes (dashed lines) were constructed using the 5th-lowest and 5th-highest $g(r)$ values of 199 Monte Carlo simulations. Solid circles indicate observed point patterns. The density of sapling aggregation peaked at the 5 m distance away from adults (D),while juveniles and adults showed closer spatial aggregation at scales below 5 m (B) than saplings and adults. Between saplings and juveniles,

they had the closest association compared to offspring-adult associations (F). The ring width was 3 m. (A), (C), (E) the distribution of each pair of life stages in the plot (green circles for adults, blue circles for juveniles, red circles for saplings).
