# Supplementary material for: The Relative Importance of Janzen-Connell Effects in Influencing the Spatial Patterns at the Gutianshan Subtropical Forest
Source: PLoS One. 2013 Sep 5;8(9):e74560. doi: 10.1371/journal.pone.0074560 (PMC3764046; doi:10.1371/journal.pone.0074560)
Supplement: Table S1 — Growth forms and abundance of 46 species and the results of analysis 1. (PDF) [file pone.0074560.s005.pdf]

Table S1. Growth forms and abundance of 46 species and the results of analysis 1. Bold font indicates that the spatial pattern of adults (Ad), juveniles (Ju), and saplings (Sa) of each species was significantly associated with the covariate, based on the Berman test under the null model of pattern reconstruction(*p*-value < 0.05).C, U, and S denote canopy trees, under-story trees, and shrubs, respectively

Species	Growth forms	Abundance	elevation					
			Z1-value			*p*-value		
			Ad	Ju	Sa	Ad	Ju	Sa
Acer cordatum	U	539	-1.81	1.43	2.33	0.06	0.15	**0.01**
Adinandra millettii	C	661	-4.01	-3.87	-4.62	**0.01**	**0.01**	**0.01**
Albizia kalkora	C	454	2.54	2.83	3.13	**0.01**	**0.01**	**0.01**
Alniphyllum fortunei	C	247	0.80	-0.19	0.42	0.49	0.88	0.66
Camellia chekiang-oleosa	U	8251	1.59	5.27	11.68	0.13	**0.01**	**0.01**
Camellia fraterna	U	4108	-1.09	-1.62	-1.97	0.32	0.09	**0.04**
Castanopsis eyrei	C	12332	0.10	3.00	5.71	0.93	**0.01**	**0.01**
Castanopsis fargesii	C	1228	0.61	-0.21	-2.44	0.58	0.81	**0.02**
Castanopsis tibetana	C	259	-2.74	-2.48	-3.49	**0.01**	**0.01**	**0.01**
Chimonanthus salicifolius	S	7795	2.25	2.38	1.72	**0.04**	**0.03**	0.11
Cleyera japonica	U	479	1.53	2.20	2.27	0.12	**0.03**	**0.01**
Corylopsis glandulifera	U	3341	4.77	8.82	5.66	**0.01**	**0.01**	**0.01**
Dalbergia hupeana	C	261	-0.55	0.24	1.59	0.65	0.88	0.11
Daphniphyllum oldhamii	C	2711	-2.37	-1.69	-3.50	**0.01**	**0.11**	**0.01**
Distylium myricoides	U	3454	-1.65	-2.09	-2.58	0.13	**0.02**	**0.01**
Elaeocarpus decipiens	C	567	-4.51	-4.49	-5.15	**0.01**	**0.01**	**0.01**
Elaeocarpus japonicus	C	228	-3.34	-2.93	-3.91	**0.01**	**0.01**	**0.01**
Eurya muricata	U	6070	0.52	2.61	1.50	0.68	**0.02**	0.15
Eurya rubiginosa var. *attenuata*	S	2762	0.84	-0.10	0.09	0.45	0.94	0.93
Ilex elmerrilliana	U	398	-3.69	-2.00	-3.48	**0.01**	**0.03**	**0.01**
Lithocarpus glaber	C	1309	-2.79	-2.78	-4.23	**0.01**	**0.02**	**0.01**
Loropetalum chinense	U	4452	-4.60	1.71	3.57	**0.01**	0.09	**0.01**
Lyonia ovalifolia var. *hebecarpa*	U	419	1.95	2.92	3.63	**0.04**	**0.01**	**0.01**
Machilus thunbergii	C	1377	-2.36	0.99	-0.74	**0.01**	0.35	0.49
Meliosma oldhamii	C	1142	1.97	2.89	3.92	**0.03**	**0.01**	**0.01**

Michelia skinnneriana	U	243	-2.98	-3.00	-2.86	**0.01**	**0.01**	**0.01**
Myrica rubra	C	906	1.86	3.06	2.55	0.07	**0.01**	**0.01**
Neolitsea aurata var. *chekiangensis*	U	9053	0.14	-1.91	-4.53	0.90	0.05	**0.01**
Photinia glabra	U	776	-2.53	-2.71	-2.31	**0.01**	**0.01**	**0.02**
Pieris formosa	U	1259	1.27	0.96	-0.06	0.23	0.40	0.97
Pinus massoniana	C	2060	3.51	3.65	3.20	**0.01**	**0.01**	**0.01**
Quercus serrata	U	3508	4.91	4.05	3.37	**0.01**	**0.01**	**0.01**
Raphiolepis indica	S	1972	-1.34	1.55	3.49	0.21	0.12	**0.01**
Rododendron latoucheae	U	2803	-3.00	-0.44	-1.15	**0.01**	0.66	0.25
Rododendron ovatum	U	10767	-1.55	3.69	5.20	0.12	**0.01**	**0.01**
Rododendron simsii	S	4792	3.28	4.99	4.98	**0.01**	**0.01**	**0.01**
Schima superba	C	8470	0.34	3.98	6.76	0.79	**0.01**	**0.01**
Styrax odoratissimus	U	535	0.69	2.49	2.05	0.54	**0.01**	0.05
Symplocos stellaris	U	626	0.72	-2.14	-3.28	0.47	**0.04**	**0.01**
Syzygium buxifolium	U	3386	-2.61	-0.60	-1.71	**0.01**	0.58	0.09
Ternstroemia gymnanthera	U	3158	-3.56	-2.97	-3.24	**0.01**	**0.01**	**0.01**
Toxicodendron succedaneum	C	335	-2.66	0.54	1.96	**0.02**	0.65	**0.04**
Vaccinium bracteatum	U	2319	-0.39	3.67	5.06	0.71	**0.01**	**0.01**
Vaccinium carlesii	U	1802	-3.02	-3.52	-2.05	**0.01**	**0.01**	**0.04**
Vaccinium mandarinorum	U	2227	1.32	4.67	5.87	0.22	**0.01**	**0.01**
Viburnum erosum	S	352	2.92	3.68	3.46	**0.01**	**0.01**	**0.01**

Species	Growth forms	Abundance	slopes					
			Z1-value			*p*-value		
			Ad	Ju	Sa	Ad	Ju	Sa
Acer cordatum	U	539	4.58	3.11	3.32	**0.01**	**0.01**	**0.01**
Adinandra millettii	C	661	-0.05	0.51	2.22	0.98	0.64	**0.04**
Albizia kalkora	C	454	-0.28	-0.64	-0.68	0.78	0.53	0.47
Alniphyllum fortunei	C	247	0.04	1.47	0.05	0.96	0.15	0.94
Camellia chekiang-oleosa	U	8251	-0.26	-0.33	0.77	0.83	0.71	0.41
Camellia fraterna	U	4108	0.98	1.48	1.81	0.30	0.15	**0.08**
Castanopsis eyrei	C	12332	0.70	0.33	0.52	0.47	0.69	0.63
Castanopsis fargesii	C	1228	0.25	-0.10	0.07	0.82	0.94	0.96
Castanopsis tibetana	C	259	0.01	-1.37	-0.98	0.99	0.17	0.35
Chimonanthus salicifolius	S	7795	3.91	5.28	6.26	**0.01**	**0.01**	**0.01**
Cleyera japonica	U	479	-0.69	0.05	-0.49	0.54	0.96	0.62
Corylopsis glandulifera	U	3341	-1.27	-0.92	-0.48	0.19	0.37	0.60
Dalbergia hupeana	C	261	0.71	2.43	1.43	0.48	**0.02**	0.12
Daphniphyllum oldhamii	C	2711	0.86	2.56	5.26	0.41	**0.01**	**0.01**
Distylium myricoides	U	3454	0.50	2.03	1.85	0.64	**0.03**	0.07
Elaeocarpus decipiens	C	567	1.15	-0.03	-0.48	0.25	0.99	0.62
Elaeocarpus japonicus	C	228	1.51	0.51	-0.06	0.13	0.58	0.96
Eurya muricata	U	6070	1.47	0.79	1.65	0.16	0.44	0.09
Eurya rubiginosa var. *attenuata*	S	2762	0.08	0.71	0.92	0.95	0.47	0.36
Ilex elmerrilliana	U	398	1.72	2.19	-0.75	0.10	**0.04**	0.45
Lithocarpus glaber	C	1309	4.64	3.50	3.94	**0.01**	**0.01**	**0.01**
Loropetalum chinense	U	4452	2.69	-0.25	-1.83	**0.01**	0.82	0.07
Lyonia ovalifolia var. *hebecarpa*	U	419	1.58	-0.17	-1.10	0.12	0.88	0.26
Machilus thunbergii	C	1377	-0.15	-1.13	-1.34	0.88	0.25	0.19
Meliosma oldhamii	C	1142	-2.25	-1.33	-2.02	**0.03**	0.21	0.05
Michelia skinnneriana	U	243	-2.50	-3.20	-3.03	**0.02**	**0.01**	**0.01**
Myrica rubra	C	906	-1.55	-0.79	0.03	0.13	0.48	0.98

Neolitsea aurata var. *chekiangensis*	U	9053	0.40	1.17	0.65	0.67	0.23	0.49
Photinia glabra	U	776	2.22	3.32	2.53	**0.03**	**0.01**	**0.01**
Pieris formosa	U	1259	2.12	1.91	1.96	**0.04**	0.07	0.06
Pinus massoniana	C	2060	-2.94	-2.67	-2.61	**0.01**	**0.02**	**0.03**
Quercus serrata	U	3508	-2.90	-2.16	-1.77	**0.01**	**0.04**	0.09
Raphiolepis indica	S	1972	2.57	-0.68	1.14	**0.02**	0.51	0.25
Rododendron latoucheae	U	2803	2.77	2.71	2.60	**0.02**	**0.02**	**0.01**
Rododendron ovatum	U	10767	5.23	3.28	2.27	**0.01**	**0.01**	**0.02**
Rododendron simsii	S	4792	-1.56	-1.83	-2.21	0.13	0.07	**0.02**
Schima superba	C	8470	1.05	-3.27	-2.03	0.30	**0.01**	**0.04**
Styrax odoratissimus	U	535	0.32	-0.33	1.01	0.70	0.74	0.34
Symplocos stellaris	U	626	-0.70	-1.83	-1.19	0.49	0.06	0.23
Syzygium buxifolium	U	3386	1.68	-1.31	-3.01	0.11	0.22	**0.02**
Ternstroemia gymnanthera	U	3158	1.14	0.55	-0.89	0.26	0.60	0.44
Toxicodendron succedaneum	C	335	1.67	0.56	3.28	0.10	0.58	**0.01**
Vaccinium bracteatum	U	2319	3.53	0.76	-0.70	**0.01**	0.40	0.51
Vaccinium carlesii	U	1802	1.96	3.24	1.90	0.05	**0.01**	0.06
Vaccinium mandarinorum	U	2227	1.00	-1.51	0.34	0.33	0.13	0.75
Viburnum erosum	S	352	0.07	-0.59	1.18	0.94	0.53	0.23

Species	Growth forms	Abundance	convexity					
			Z1-value			*p*-value		
			Ad	Ju	Sa	Ad	Ju	Sa
Acer cordatum	U	539	-2.77	0.92	0.82	**0.01**	0.38	0.43
Adinandra millettii	C	661	-5.26	-3.42	-3.56	**0.01**	**0.01**	**0.01**
Albizia kalkora	C	454	2.55	3.73	3.93	**0.01**	**0.01**	**0.01**
Alniphyllum fortunei	C	247	0.32	1.54	-1.46	0.78	0.12	0.15
Camellia chekiang-oleosa	U	8251	-1.85	0.84	1.68	0.05	0.41	0.12
Camellia fraterna	U	4108	-5.00	-5.59	-6.20	**0.01**	**0.01**	**0.01**
Castanopsis eyrei	C	12332	7.03	5.07	3.15	**0.01**	**0.01**	**0.01**
Castanopsis fargesii	C	1228	-2.44	-3.67	-6.02	**0.02**	**0.01**	**0.01**
Castanopsis tibetana	C	259	-4.98	-3.00	-4.84	**0.01**	**0.01**	**0.01**
Chimonanthus salicifolius	S	7795	0.97	1.84	2.15	0.31	0.07	0.05
Cleyera japonica	U	479	-1.59	1.83	2.25	0.13	0.08	**0.02**
Corylopsis glandulifera	U	3341	3.68	4.90	4.54	**0.01**	**0.01**	**0.01**
Dalbergia hupeana	C	261	-5.53	-4.07	-0.40	**0.01**	**0.01**	0.75
Daphniphyllum oldhamii	C	2711	-2.07	-1.81	-3.63	**0.04**	0.08	**0.01**
Distylium myricoides	U	3454	-6.28	-3.76	-4.43	**0.01**	**0.01**	**0.01**
Elaeocarpus decipiens	C	567	-1.57	-3.62	-3.10	0.13	**0.01**	**0.01**
Elaeocarpus japonicus	C	228	-4.03	-3.88	-4.62	**0.01**	**0.01**	**0.01**
Eurya muricata	U	6070	0.39	3.89	3.07	0.72	**0.01**	**0.02**
Eurya rubiginosa var. *attenuata*	S	2762	-2.57	-2.14	-3.34	**0.02**	0.05	**0.01**
Ilex elmerrilliana	U	398	-4.07	-2.35	-5.35	**0.01**	**0.01**	**0.01**
Lithocarpus glaber	C	1309	-1.40	0.86	-3.26	0.19	0.37	**0.01**
Loropetalum chinense	U	4452	-5.87	4.35	5.31	**0.01**	**0.01**	**0.01**
Lyonia ovalifolia var. *hebecarpa*	U	419	0.44	2.72	2.83	0.70	**0.01**	**0.01**
Machilus thunbergii	C	1377	-7.59	-4.85	-4.11	**0.01**	**0.01**	**0.01**
Meliosma oldhamii	C	1142	0.73	3.63	4.95	0.47	**0.01**	**0.01**
Michelia skinnneriana	U	243	-4.40	-3.91	-3.35	**0.01**	**0.01**	**0.01**
Myrica rubra	C	906	5.85	4.65	3.51	**0.01**	**0.01**	**0.01**

Neolitsea aurata var. *chekiangensis*	U	9053	-2.03	-3.21	-5.17	0.06	**0.01**	**0.01**
Photinia glabra	U	776	-3.43	-2.28	-3.01	**0.01**	**0.04**	**0.01**
Pieris formosa	U	1259	-1.88	0.44	-0.82	0.06	0.68	0.42
Pinus massoniana	C	2060	6.67	3.74	2.75	**0.01**	**0.01**	**0.01**
Quercus serrata	U	3508	6.19	3.57	2.05	**0.01**	**0.01**	**0.03**
Raphiolepis indica	S	1972	2.22	3.70	3.66	**0.03**	**0.01**	**0.01**
Rododendron latoucheae	U	2803	-5.77	-1.08	0.35	**0.01**	0.29	0.72
Rododendron ovatum	U	10767	-3.23	5.74	6.07	**0.01**	**0.01**	**0.01**
Rododendron simsii	S	4792	5.40	5.29	4.75	**0.01**	**0.01**	**0.01**
Schima superba	C	8470	5.53	4.09	4.35	**0.01**	**0.01**	**0.01**
Styrax odoratissimus	U	535	1.24	4.38	4.43	0.21	**0.01**	**0.01**
Symplocos stellaris	U	626	2.70	-1.39	-3.27	**0.03**	0.14	**0.01**
Syzygium buxifolium	U	3386	0.13	3.63	3.93	0.92	**0.01**	**0.01**
Ternstroemia gymnanthera	U	3158	-2.81	2.53	2.67	**0.01**	**0.01**	**0.01**
Toxicodendron succedaneum	C	335	-3.49	1.43	2.15	**0.01**	0.16	**0.03**
Vaccinium bracteatum	U	2319	-0.83	4.37	6.34	0.41	**0.01**	**0.01**
Vaccinium carlesii	U	1802	-5.48	-1.55	-0.15	**0.01**	0.14	0.90
Vaccinium mandarinorum	U	2227	-0.35	4.50	4.72	0.72	**0.01**	**0.01**
Viburnum erosum	S	352	1.05	2.92	2.65	0.27	**0.01**	**0.03**
